# Success in the Dental Profession

**Published:** 1862-11

**Authors:** C. C. Dills


### SUCCESS IN THE DENTAL PROFESSION.

BY C. C. DILLS, D. D. S.

### NO. 4.—FRANKNESS; HONESTY; TRUTH.

"Truth loves open dealing."—SHAKSPEARE.

"Fear not my truth; the moral of my wit
Is—plain and true,—there's all the reach of it."
SHAKSPEARE.

"And is it then altogether impossible to take up one's abode with truth, and to let all sweet, homely feelings grow about it and cluster around it, and to smile upon it as on a kind father or mother, and to sport with it, and to hold light and merry talk with it, as with a loved brother or sister, and to fondle it and play with it as with a child?"—ANON.

UPON the whole, I am somewhat surprised that there really exists no call for dissembling in the dental profession,—others have so much thought there did; but that, on the contrary, there is a premium attending the plain truth. I confess too, in this connection, I sometimes wish for a return of the days when I was "a little too confiding," and the incident frankness thereto. I was trusted freely then, though my inexperience was evident. For do I not know to-day, that as I approximate a healthy simplicity, and show myself identified with my patients' interest—as I may truthfully do so many times—that then most am I trusted, and is work heaped upon me without inventory!—because now my patient and I have said, "Let there be truth between us two forevermore."

Truth has never yet been received as a policy of action in the business world; it affords, therefore, a new, and it is reasonable to suppose, a profitable field of action. I remember reading somewhere, in fiction or fact, of a tremendous success of a business house, by the adoption of absolute truth in all its dealings. This read natural enough to be real, yet I fear it was not; but whatever the fact may be in the business world generally, I vouch that this view of the case may be verified any time in the dental profession,—the mystic character of which, favoring artifice heretofore, favors honesty to-day the more.

Does there occupy my chair some unfortunately suspicious and close-fisted patient, feeling that now, in spite of himself, he will have to pay an almighty dollar a piece for an indefinite number of holes drilled through solid bones, and filled? Let us see the workings of frank honesty in such a case. In examining his teeth, I have occasion to say, here is a small cavity, but it need not be filled just now;—and here is another, and another, but your teeth are of a good texture, decay slowly, and you have only to consult some honest dentist once a year, and all's well;—but here is one, and there is one, and there another, that must be filled at once;—and do I not fill them, at my own price, he, all changed about now, insisting that I fill all that need it, and assuring me that he likes the way I talk, and that he will certainly select me as the honest dentist to be consulted hereafter?

Does another such person in character apply to me for artificial teeth, feeling that as he has lost his natural teeth rapidly heretofore, so he will in the future; and that, therefore, economy would dictate that he sacrifice some remaining good teeth, and have an entire set? Let me treat him with frank honesty. Sir, you don't understand *dental* economy. Though you have lost your teeth rapidly heretofore, you will not in the future; on the contrary, such teeth of yours as have withstood decay thus far in life will, in all probability, never decay at all. I can do the best thing possible for you at half the supposed cost. Is not this man now my friend and patron forever?—and do I not make up an hundred fold in the end, for this greater profit denied myself just now? I certainly do; I have watched it—*honesty* "pays" in dentistry throughout.

Again: Does some meddlesome, envious or curious patient see fit to quiz me concerning my profits? I will not be surprised or disconcerted. Granting the facts he presumes to be true, I give him more. I may even grant a similarity of profits between ours and other professions,—what then? do we not render the same satisfactory equivalent for that we receive?—in fact, do we not render a higher equivalent than most any other profession?

Then again, I appeal from our profit to our salary,—does that average higher with us than with others similarly deserving?—or, *do our circumstances, as a class, show our profits too high*? Even as "Honesty is the best policy," so do these facts disconcert my opponent.

But thus far I have only spoken of truth as a business policy; let me now speak of it as a principle. It is only when truth is received and practiced as a *principle*, that we get its full benefit and beauty; then it makes *men* of us, as well as money for us. Our profession combines peculiarly social and business life, and he who would succeed among us must neglect neither the one nor the other. To speak of truth as a principle, then, will be to apply it to our social life. And the greatest significance I can give to truth is to give *purity* as its synonym. Now, there is that beauty in a pure character—that application of it to every phase of life—it so enhances life—it so delights one to associate with it—so compels one to render homage to it—it is so commendable, and so rare a thing—and yet one would think so attainable, that I feel to properly write upon it would be to make every one fall in love with it, and go about attaining it. We are all attracted by it, then why not attract with it? Why not invite patronage as well through our character, as our work or the newspaper? I tell you it is as effectual, and a perpetual source of gratification and self-assurance besides. Then, as surely, too, as a pure character is attractive and self-assuring, so an impure one is repulsive and self-distrusting. How sickly, sycophantic, and unsightly is that twist and wrangle of the face, and that debasing distortion of nature, so necessary with the pretender. Most truly did Socrates say, "The shortest and surest way to live with honor in the world is to be in reality what you would appear to be."

We all recognize these things as true, then why not act upon them in every particular? I am ashamed at the cheapness we hold our integrity, at the liberty we give our patients to gainsay us. Too many of them embrace this liberty, and too freely; yet I trace it to the general consent and invitation of the dentist. He says it will not hurt to excavate or extract a tooth, and expects to be contradicted. He is not particular in his regard for truth—how can his patient be in crediting him with it?

It has ever been a fancy with me to have the privilege of *drilling* a set of patients,—in *truth* on my part, and *confidence* on theirs. This may ever remain a fancy, but I will not be deterred from my honesty, or discouraged from my purpose;—the occasional success is encouragement enough. Aside from the moral satisfaction of such a relation of dentist and patient, let us look at its practical workings in the one operation of extracting teeth. We all know that in nine cases out of ten, the patient overrates the pain to be inflicted by this operation; and in many cases vastly overrates it,—so much so, that could he by any means anticipate the real amount, he would not regard its infliction. Now, it is just here that the dentist wants to assert a *fact*, and be *believed;* and if his integrity be already established with his patient, he can do so, with the most satisfactory results,—otherwise he can not. His most earnest and honest appeals, in the absence of this established *strict truth*, are utterly unheeded by the patient, or met with a flat contradiction. Now, let any one notice these things, and he will be surprised, and, I trust, chagrined at the result. Then let us *be firm* in *absolute truth*—with the child and with the adult—with the unimportant and the important thing alike—that thus this virtue may become a fixed fact and habit of our life. It will strengthen by practice, giving a nobleness, integrity and self-possession of character—so beautiful that one would court it for that alone, even though he denied virtue to the practice of the virtues.

I but poorly express myself here, and fall far short of the importance and bearing of my subject, however unimportant and irrelevant this may be considered to be. I really feel that all other policies of business, and traits of character, in comparison with that of simple, straightforward integrity, are utterly insignificant. I can but close, then, in the language of Franklin—"*Let honesty be as the breath of thy soul,* and never forget to have a penny when all thy expenses are enumerated and paid; then shalt thou reach the point of happiness, and independence shall be thy shield and buckler, thy helmet and crown; then shall thy soul walk upright, nor stoop to the silken wretch because he hath riches, nor pocket an abuse because the hand which offers it wears a ring set with diamonds."

